# Photoactive Yellow Protein Represents a Distinct, Evolutionarily Novel Family of PAS Domains

**DOI:** 10.1128/jb.00300-22

**Published:** 2022-10-05

**Authors:** Jiawei Xing, Vadim M. Gumerov, Igor B. Zhulin

**Affiliations:** a Department of Microbiology, The Ohio State University, Columbus, Ohio, USA; b Translational Data Analytics Institute, The Ohio State University, Columbus, Ohio, USA; NCBI, NLM, National Institutes of Health

**Keywords:** photoreceptor, bacterial signal transduction, computational biology, phylogenetics, protein evolution, Per-Arnt-Sim domain, PAS domain, photoactive yellow protein, PYP, *p*-coumaric acid, pCA, evolution, photoreceptors

## Abstract

Photoactive yellow protein (PYP) is a model photoreceptor. It binds a *p*-coumaric acid as a chromophore, thus enabling blue light sensing. The first discovered single-domain PYP from Halorhodospira halophila has been studied thoroughly in terms of its structural dynamics and photochemical properties. However, the evolutionary origins and biological role of PYP homologs are not well understood. Here, we show that PYP is an evolutionarily novel domain family of the ubiquitous PAS (Per-Arnt-Sim) superfamily. It likely originated from the phylum *Myxococcota* and was then horizontally transferred to representatives of a few other bacterial phyla. We show that PYP is associated with signal transduction either by domain fusion or by genome context. Key cellular functions modulated by PYP-initiated signal transduction pathways likely involve gene expression, motility, and biofilm formation. We identified three clades of the PYP family, one of which is poorly understood and potentially has novel functional properties. The Tyr42, Glu46, and Cys69 residues that are involved in *p*-coumaric acid binding in the model PYP from H. halophila are well conserved in the PYP family. However, we also identified cases where substitutions in these residues might have led to neofunctionalization, such as the proposed transition from light to redox sensing. Overall, this study provides definition, a newly built hidden Markov model, and the current genomic landscape of the PYP family and sets the stage for the future exploration of its signaling mechanisms and functional diversity.

**IMPORTANCE** Photoactive yellow protein is a model bacterial photoreceptor. For many years, it was considered a prototypical model of the ubiquitous PAS domain superfamily. Here, we show that, in fact, the PYP family is evolutionarily novel, restricted to a few bacterial phyla and distinct from other PAS domains. We also reveal the diversity of PYP-containing signal transduction proteins and their potential mechanisms.

## INTRODUCTION

Photoreceptors are light-sensitive proteins that mediate light-induced signal transduction in numerous living organisms (1). The biological functions modulated by photoreceptors include phototropism (2), stress responses (3), circadian rhythms (4), and development (5). The best-studied model photoreceptors include rhodopsin, green fluorescent protein (GFP), phytochrome, and photoactive yellow protein (PYP) (6). Although GFP has been widely used for cell labeling, limitations exist due to its bulky size (238 amino acid residues; 27 kDa) and oxygen requirement (7). PYP is a much smaller (125 amino acid residues; 14 kDa), stable, soluble protein (8), which has emerged as a promising probe for protein labeling (9) and as a photoswitchable regulator for protein activities (10), with applications in real-time cell imaging (11), DNA methylation labeling (12), and optogenetic transcription regulation (13).

**FIG 1:**
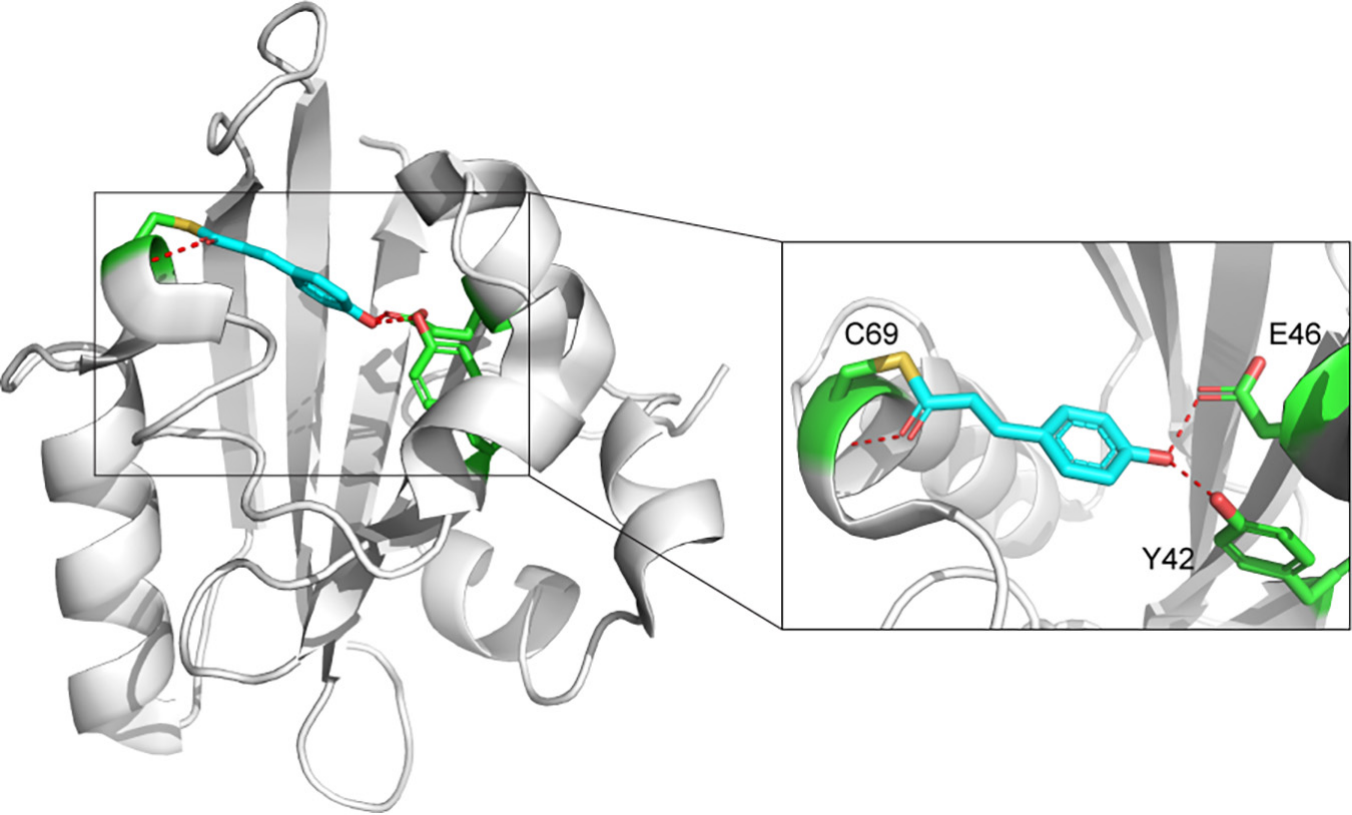
Structure of PYP and its conserved chromophore-binding residues. Shown is the structure of *H. halophila* PYP with *p*-coumaric acid (PDB accession number 1NWZ). Cyan, *p*-coumaric acid; green, conserved residues for cofactor binding; yellow stick, thioester bond with C69; red dashes, hydrogen bonds with Tyr42, Glu46, and Cys69.

PYP was originally found in the halophilic purple bacterium Halorhodospira halophila (8). It has a putative function as a light sensor in negative phototaxis (14); however, its downstream partner protein was never identified. PYP binds *p*-coumaric acid (pCA) as a chromophore and detects blue light with an absorbance maximum at 446 nm (8). Three key residues are involved in chromophore binding by H. halophila PYP. Cys69 forms a covalent thioester bond with pCA, whereas Tyr42 and Glu46 form hydrogen bonds with the phenolate oxygen of pCA (15–17) (Fig. 1). The photocycle of PYP can be summarized as follows. Upon blue light illumination, pCA undergoes *trans*-to-*cis* isomerization (18), resulting in a red-shifted state (pR) (19). Glu46 then donates a proton to pCA (20), causing conformational changes and partial unfolding of the protein (21, 22). This leads to a blue-shifted state (pB), which is postulated to be the signaling state when PYP transduces signals to its unknown partners (23). Finally, pCA reisomerizes to recover the initial ground state (pG), completing the reversible photocycle of PYP (24, 25).

PYP is a single-domain protein, and it was proposed as the structural prototype for the ubiquitous sensor Per-Arnt-Sim (PAS) domain superfamily (26–28). PAS domains have a common structural fold exemplified by PYP (29, 30), and they bind various ligands and cofactors (31, 32). The vast majority of PAS domains are found in multidomain signaling proteins, such as sensor histidine kinases, chemoreceptors, and transcription factors, thus serving as sensors in signal transduction pathways (33, 34). A great number of studies have been conducted leveraging PYP as a model (35–38), producing a thorough understanding of its structural dynamics (39) and photochemical properties (27, 28). However, the signaling partners and biological outputs of PYP homologs are poorly understood. Recent studies show that PYP homologs exhibit great functional diversity across organisms (40–47), and *H. halophila* PYP is just one example of a potentially rich family (47). To better understand the diversity of this group of proteins, we conducted a comprehensive comparative genomic analysis of PYP and its homologs. We now formally define the PYP protein family based on sequence similarity and the conservation of chromophore-binding residues in the context of structure. Phylogenetic analyses revealed that PYP is an evolutionarily novel family of the PAS superfamily.

## RESULTS

### Identification of PYP homologs.

We used three independent approaches to identify PYP homologs: (i) Markov clustering (48) with PAS family protein sequences from the Pfam database (49), (ii) BLAST searches (50) against the NCBI Reference Sequence (RefSeq) database (51), and (iii) HMMER searches (52) against the TIGRFAM database models (53).

**FIG 2:**
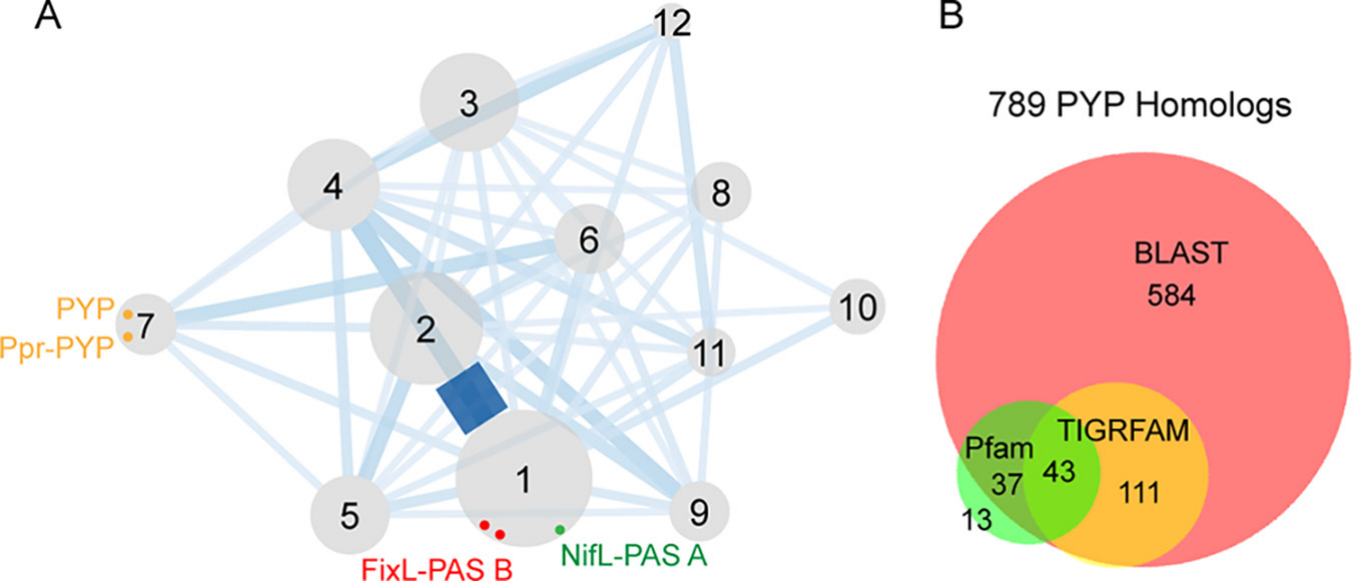
Identification of PYP homologs. (A) Network of the PAS family (Pfam entry PF00989; InterPro entry IPR013767) protein clusters. Other families from the PAS superfamily (Pfam entry CL0183; InterPro entry IPR035965) were not included. Clusters were formed by the Markov cluster algorithm with an inflation parameter value of 1.4. Clusters are numbered from 1 to 12 according to their sizes (from large to small). The distances between clusters and the widths of the edges are calculated based on the numbers of observed BLAST hits divided by the number of all possible BLAST hits between clusters (see Materials and Methods). Dots within clusters show PAS domains with known cofactors and structures: PYP from Halorhodospira halophila binds *p*-coumaric acid (yellow) (PDB accession number 1NWZ), Ppr-PYP from Rhodospirillum centenum binds *p*-coumaric acid (yellow) (PDB accession number 1MZU), FixL-PAS B from Bradyrhizobium japonicum binds heme *b* (blue) (PDB accession number 1DP9), FixL-PAS B from Sinorhizobium meliloti binds heme *b* (blue) (PDB accession number 1EW0), and NifL-PAS A from Azotobacter vinelandii binds flavin adenine dinucleotide (FAD) (green) (PDB accession number 2GJ3). (B) Numbers of PYP homologs identified using three independent approaches.

**(i) PYP homologs are found in a distinct cluster within the Pfam PAS family.** In the authoritative protein family database Pfam, *H. halophila* PYP (NCBI accession number WP_011814604.1; INSDC accession number ABM62582.1) is found in the PAS family (Pfam entry PF00989; InterPro entry IPR013767), the largest family of the PAS superfamily (see Table S1 in the supplemental material) (49). To identify PYP homologs and their distribution within this family, we retrieved all PAS domain sequences comprising this family from the Pfam database. By running the Markov cluster algorithm (inflation [*I*] = 1.4), we identified 12 clusters of protein sequences (see Materials and Methods). Intriguingly, while most PAS domains with different ligand-binding functions were assigned to the largest cluster (cluster 1), PYP homologs were found exclusively in a smaller cluster containing 93 sequences (cluster 7) (Fig. 2A). The cluster included *H. halophila* PYP and Rhodospirillum centenum Ppr, a PYP/bacteriophytochrome/histidine kinase hybrid protein (54). Most sequences (>80%) within cluster 7 contain conserved Tyr42, Glu46, and Cys69 residues, indicating a conserved binding site for pCA (Fig. 1). This result suggests that PYP homologs have distinct sequences compared to those of other ligand-binding PAS domains.

To determine the robustness of the PYP cluster, we tested a set of different inflation values. This parameter controls the granularity of clustering so that lower inflation values generate larger clusters (48). An inflation parameter value of 1.3 gave a result similar to that with an *I* value of 1.4: 10 clusters were formed, and all PYP homologs were grouped into a single, small cluster (Table S2). Inflation values of 1.2 and 1.0 failed to classify the PAS family into meaningful clusters: an *I* value of 1.2 resulted in one large cluster containing more than 95% of the sequences, and an *I* value of 1.0 failed to converge sequences into stable clusters (Table S2). Therefore, 1.3 is the lowest meaningful inflation value for the PAS family, and the PYP cluster is still stable and well separated from other clusters under this condition. Moreover, we established that higher inflation values (*I* = 2 and *I *= 6) also grouped PYP homologs into a separate cluster without breaking down into smaller clusters (Table S2), which further demonstrates that the PYP cluster is stable.

Many well-studied ligand/cofactor-binding PAS domains are present in other PAS families (Table S3). To compare PYP with these PAS domains, we conducted all-versus-all BLAST analysis using well-studied PAS domains combined with PYP homologs from cluster 7. The results showed that PYP homologs form a separate group distinct from other PAS domains (Fig. S1A). PYP contains an array of phenylalanines and tyrosines located near the chromophore-binding site (Fig. S1B and C), which were shown to regulate the absorption spectrum, thermal stability, and the photocycle (55). These highly conserved and distinct residues, together with the three pCA-binding residues, distinguish PYP from other PAS domains.

**(ii) PYP homologs identified in the RefSeq database are present in more than one Pfam family.** Our initial analysis of PYP was limited to the PAS family in Pfam (Table S1). To collect an extended set of PYP homologs, we carried out BLAST searches against RefSeq, using PYPs from multiple bacterial phyla as queries (see Materials and Methods). Altogether, 775 sequences similar to PYP were obtained. An HMMER search (52) against the Pfam-A profile database was used for domain identification. Surprisingly, while 70% of the sequences matched the PAS family, the remaining 30% of the sequences mostly matched the PAS_4 family (Pfam entry PF08448; InterPro entry IPR013656), the second largest family in the PAS superfamily (Pfam entry CL0183; InterPro entry IPR035965) (Table S1) (49). Notably, irrespective of the protein family to which they belong, more than 78% of these sequences contained conserved Tyr42, Glu46, and Cys69 residues for pCA binding (Fig. 1). The fact that PYP homologs are classified by Pfam into more than one protein family suggests that the current classification of PAS domains needs improvement.

**(iii) The HMM profile from TIGRFAM matches a small subset of PYP homologs.** TIGRFAM (53) is a protein family database that contains a profile hidden Markov model (HMM) for PYP, TIGR02373. This profile matched 155 PYP homologs in RefSeq (51), more than 97% of which contain conserved Tyr42, Glu46, and Cys69 residues for cofactor binding. The low coverage (20% of PYP homologs identified by BLAST analysis) of this profile suggests that more PYP homologs need to be identified, possibly by using an improved HMM profile.

**FIG 3:**
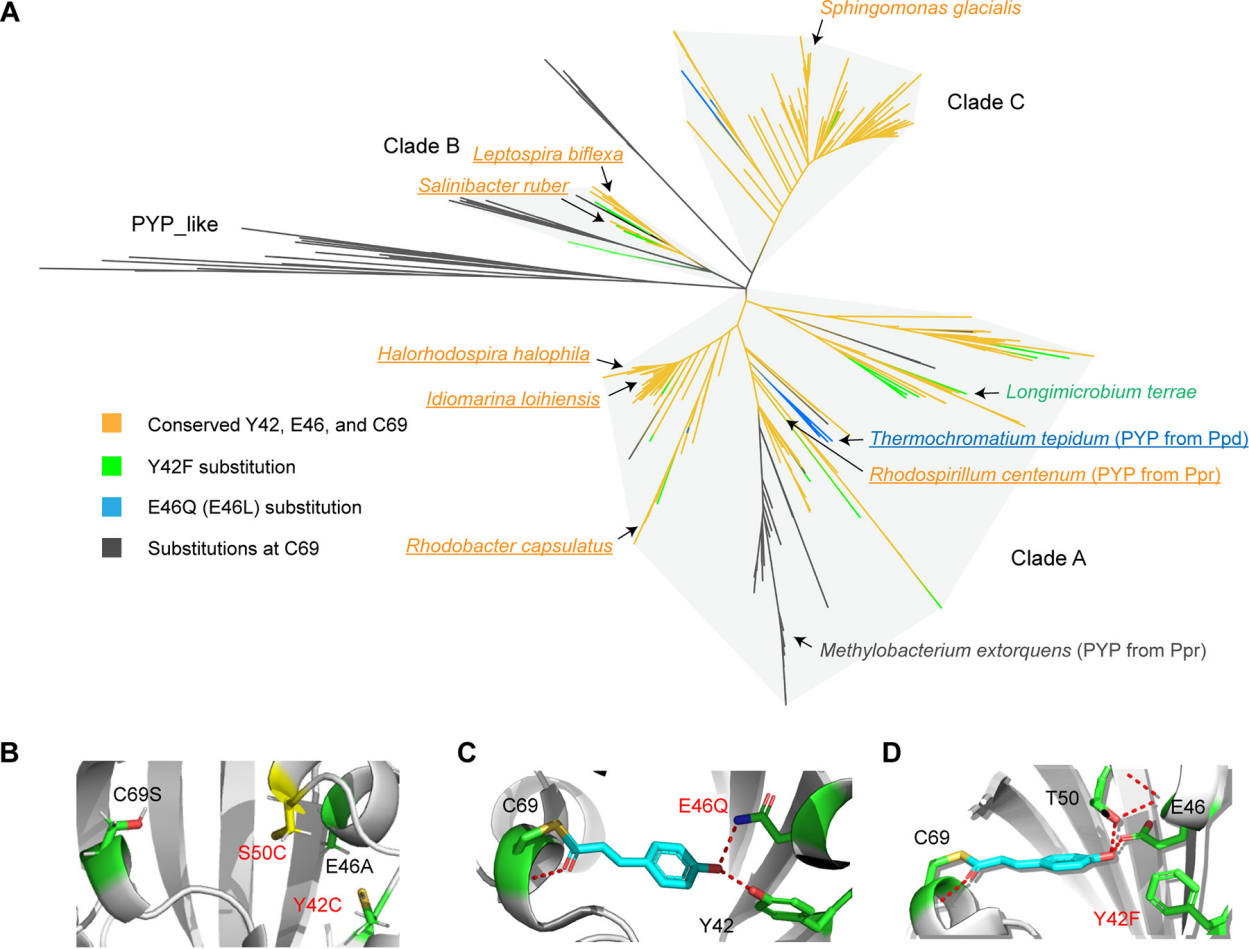
Clades of PYP homologs and conservation of key residues for chromophore binding. (A) Maximum likelihood tree of PYP homologs. PYP homologs from representative species are marked on the tree. Biochemically characterized PYPs are underlined. Orange, PYPs with invariable Tyr42, Glu46, and Cys69 residues; green, PYP with a Y42F substitution; blue, PYP with an E46Q or E46L substitution; gray, PYP with Cys69 substitutions. (B) AlphaFold structural model of methylobacterial Ppr-PYP showing key substitutions. (C) Structure of *H. halophila* PYP with an E46Q mutation (PDB accession number 1UGU). (D) Structure of *H. halophila* PYP with a Y42F mutation (PDB accession number 1F9I).

In sum, 789 PYP homologs were identified using three independent approaches (Fig. 2B; Data Sets S1 and S2). This set of sequences forms three clades on a phylogenetic tree (Fig. 3A): clade A contains PYP homologs mostly from the *Proteobacteria* and *Myxococcota*, clade B contains PYP homologs mostly from the *Spirochaetota* (especially *Leptospira*) and *Bacteroidota* (especially *Salinibacter*), and clade C contains PYP homologs mostly from the *Proteobacteria* (especially *Burkholderiales*). We found that PYP homologs that are located close to each other on the tree have similar photochemical properties (Fig. 3A). For example, PYP from Idiomarina loihiensis has an absorbance maximum, pCA pK_a_, proton transfer, and pB lifetime very similar to those of *H. halophila* PYP (42); Ppd-PYP from Thermochromatium tepidum is kinetically the most similar to Ppr-PYP from R. centenum (56); and PYPs from Leptospira biflexa and Salinibacter ruber exhibit a novel photocycle state (pUV) that is not found in clade A (44, 47). Notably, no representatives from clade C have been characterized previously. Here, we compared the sequence conservation of proteins from clades A, B, and C (Fig. S2). Remarkably, clade C lost the conservation of Phe92 and Phe96, mutations of which in *H. halophila* PYP slow down the photocycle (55); it also lost the conserved residue Trp119, which is critical for UV-B absorbance (57). Interestingly, clade C contains a highly conserved Met100 residue, which was proposed to catalyze pCA reisomerization, but the distance between pCA and Met100 in the structure also needs to be considered (40). All of these findings suggest that PYP has great variation in its photodynamics, and more studies on clade C will provide a better understanding of the structural and photochemical properties of the proposed PYP family.

### Residues involved in pCA binding define the PYP family.

**FIG 4:**
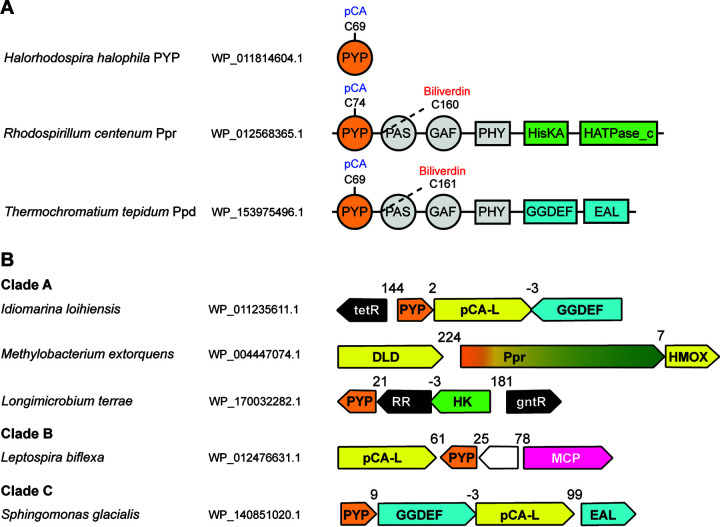
PYP association with signal transduction proteins and enzymes involved in chromophore biosynthesis and disulfide bond catalysis. (A) Domain architectures of representative PYP-containing proteins. Ppr is a fusion of PYP, a bacteriophytochrome (PHY domain), and a histidine kinase (HisKA and HATPase_c domains). Ppd is a fusion of PYP, a bacteriophytochrome (PHY domain), and a diguanylate cyclase/phosphodiesterase (GGDEF and EAL domains). Key residues for chromophore binding are shown. In Ppr and Ppd, a cysteine from the N-terminal PAS domain extends into GAF for biliverdin binding. (B) Gene neighborhood of representative PYP homologs. Distances between genes are shown in base pairs. Negative distances represent overlaps between genes. Color-code for gene function: orange, PYP genes; yellow, genes related to the sensory function of PYP; black, transcriptional regulators; blue, genes related to c-di-GMP regulation; green, histidine kinase; magenta, chemoreceptor; white, hypothetical protein. Abbreviations: pCA-L, pCA-CoA ligase; DLD, dihydrolipoamide dehydrogenase; HMOX, heme oxygenase; RR, response regulator; HK, histidine kinase; MCP, methyl-accepting chemotaxis protein; GGDEF, c-di-GMP cyclase; EAL, c-di-GMP phosphodiesterase.

There are three key residues in *H. halophila* PYP involved in pCA binding (43): Cys69 forms a thioester bond and is indispensable for pCA binding (58, 59), and Tyr42 and Glu46 form hydrogen bonds and control the photocycle (60) (Fig. 1). Here, we performed a sequence conservation analysis focusing on these three conserved residues. Substitutions of the most important residue, Cys69, were found in clades A and B and in a group of distant homologs (PYP_like) (Fig. 3A). In PYP, such mutations significantly impair pCA binding and the light-sensing function (58). Many PYP sequences with Cys69 substitutions are from homologs of the multidomain histidine kinase Ppr encoded in the genomes of methylobacteria (Fig. 4A). Notably, some of these Ppr-PYPs also have Y42C and S50C substitutions (Fig. S3). This generates a potential site for a disulfide bond (43) (Fig. 3B), suggesting novel functions such as redox sensing. However, the thiol groups of two cysteines are 6 Å away from each other, which might be too far away to form a disulfide bond (usually 2 to 3 Å). Possible explanations include structure dynamics that bring two cysteines together or mixed disulfide bonds with other chemicals such as glutathione. Molecular docking using Ppr-PYP from *Methylobacterium* sp. strain Leaf123 (NCBI accession number WP_056199890.1) failed to show pCA binding (data not shown), further demonstrating its loss of pCA binding and neofunctionalization.

We found that mutations at the Glu46 position are relatively rare (<3%), with E46Q being the most common case. In *H. halophila* PYP, E46Q leads to weak hydrogen bond formation (Fig. 3C) (61), impaired proton donation (22), a red-shifted absorbance (60), and a faster photocycle (62) (but slower blue-shifted state formation [63]). In our data set, most E46Q mutations were found in clade A (Fig. 3A), which contains PYP from Ppd, a PYP/bacteriophytochrome/GGDEF protein (Fig. 4A) (56). This narrow distribution suggests that Glu46 is critical for classical PYP function, and Ppd-PYP with its E46Q substitution may represent subfunctionalization of the light sensor.

We found that the most common mutation of Tyr42 is Y42F. Y42F in *H. halophila* PYP causes changes in hydrogen bonding, shifting from Tyr42-pCA bonding to Thr50-pCA bonding (Fig. 3D), and a red shift of the absorbance maximum (64). Approximately 9% of PYP homologs have Y42F mutations that are distributed across multiple branches of clades A and B (Fig. 3A), indicating that mutation of Tyr42 is more tolerable than that of Glu46.

Taken together, 665 out of 789 PYP homologs (84%) have conserved Cys69 and are expected to bind pCA. We defined these PYP homologs as members of the PYP family and built a profile HMM for this set of sequences (see Materials and Methods). The newly built HMM, which we termed PAS_PYP, identified 744 PYP homologs in RefSeq and 218 PYP homologs in the Reference Proteomes databases (Data Set S5). This expands the current number of PYP homologs in RefSeq by more than 4-fold. PYP homologs newly identified by the PAS_PYP model are often misannotated in RefSeq as a “hypothetical protein” or a “phosphonate transporter,” etc. (Data Set S3).

The new profile HMM was built based on a small group of key sequences called seed sequences (see Materials and Methods). The seed sequences defining the PAS family in the Pfam database incorrectly incorporate PYP into this large family, while our seed sequences contain PYP homologs only (Data Set S4). Therefore, the new profile HMM matches only PYP homologs and, thus, has a higher specificity. On the other hand, the seed sequences of the TIGRFAM model include only four PYP homologs, while we used a large and balanced data set, which resulted in eight seed sequences (Fig. 2B; Data Set S4) and a model with increased sensitivity.

### PYP serves as the input for various signal transduction proteins.

Previous studies reported an association between PYP and pCA biosynthesis (43, 65, 66). Tyrosine ammonia lyase (TAL) synthesizes pCA from tyrosine (67), and pCA:CoA ligase (pCL) promotes the formation of the thioester bond between pCA and Cys69 (65, 66). Consistently, we found that at least 10% of genes encoding functional PYPs (defined by the conserved Cys69 residue) are adjacent to TAL genes (within the distance of five genes), and at least 30% are adjacent to genes coding for pCL (Data Set S3). The lower number of TAL gene neighbors may be due to pCA synthesis through other pathways, including histidine ammonia lyase and phenylalanine ammonia lyase (43). We also found that when PYP is merged with bacteriophytochrome (as seen in Ppr and Ppd), which binds a biliverdin (Fig. 4A), the gene neighborhood always contains a heme oxygenase for biliverdin synthesis (Fig. 4B; Data Set S3). Intriguingly, we found that the Ppr-PYP gene in methylobacteria is always in the same operon with a gene coding for dihydrolipoamide dehydrogenase (DLD) (Fig. 4B; Data Set S3). DLD is the E3 unit of the pyruvate dehydrogenase complex, which forms a disulfide bond and converts dihydrolipoamide to lipoamide (68). We found that this DLD near Ppr is a paralog of E3 in the same methylobacterial genome. Key residues for disulfide bond catalysis (C43 and C48) are conserved in this DLD. Therefore, this DLD paralog may be recruited by Ppr and facilitate the formation of disulfide bonds through an unknown mechanism. This further reinforces the idea of putative redox sensing in some methylobacterial Ppr-PYPs due to Y42C and S50C mutations (Fig. 3B).

PYPs are found mostly as single domain proteins, and how they transduce signals to their downstream partners is still unknown. Some studies found that a single-domain PYP binds signaling proteins that are encoded in the gene neighborhood (42, 43, 46, 65). Consequently, we analyzed gene neighborhoods for all identified PYP homologs (Fig. 4B; Data Set S3). We found that PYPs from clade A are often adjacent to various signal transduction proteins, including transcriptional regulators, histidine kinases, and diguanylate cyclases (Fig. 4B; Data Set S3). PYPs from clade B are frequently found adjacent to a chemoreceptor that lacks sensory domains (Fig. 4B; Data Set S3). This points to the potential function of PYP in phototaxis, as previously reported for *H. halophila* (14). PYPs from clade C were often found adjacent to cyclic diguanylate cyclases (GGDEF-containing proteins) (Fig. 4B; Data Set S3), suggesting a potential function in light-regulated biofilm formation.

### PYP is an evolutionarily novel PAS domain family.

**FIG 5:**
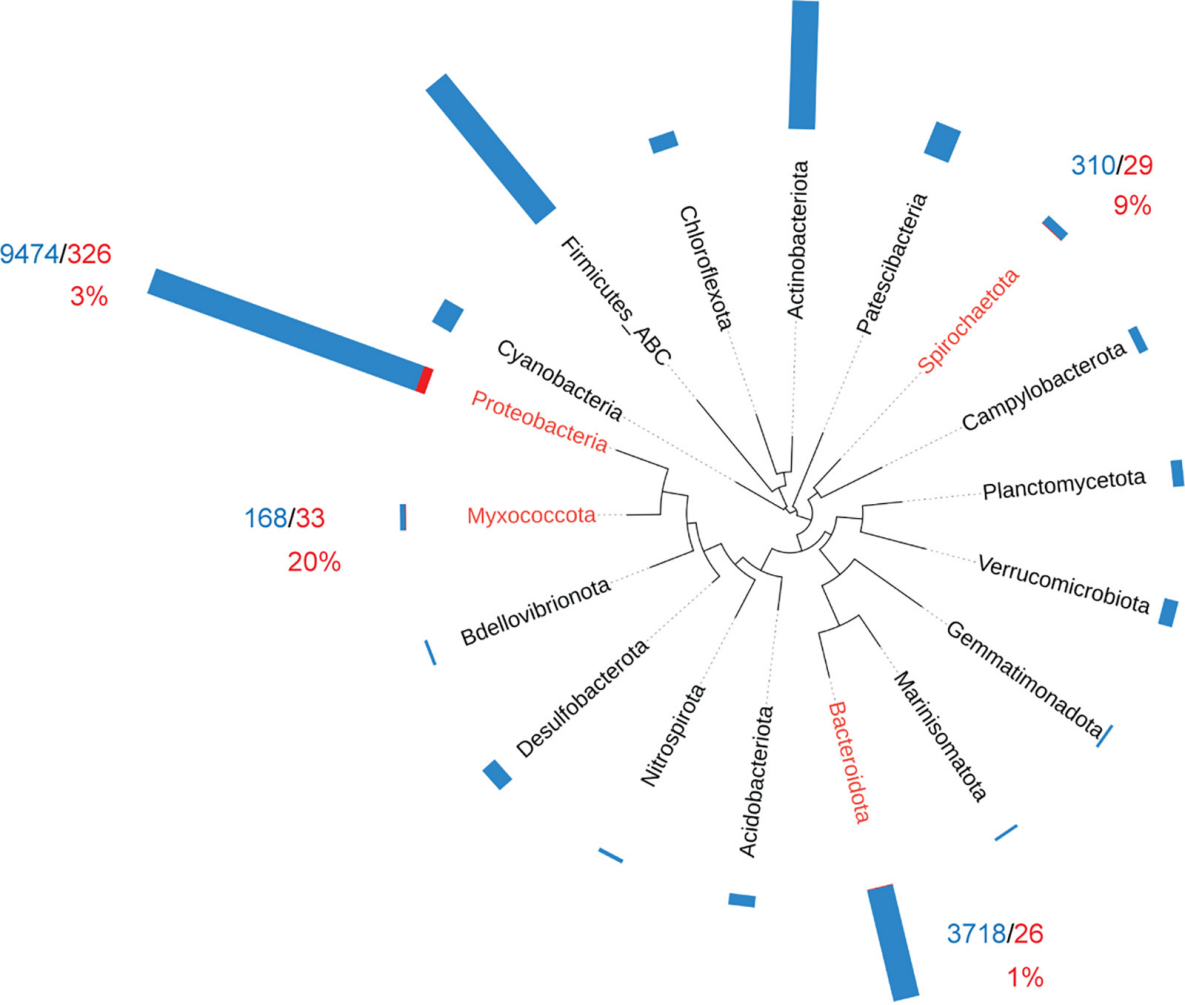
PYP phyletic distribution. Bacterial phyla with at least 10 available genomes are shown. A modified bacterial tree of life downloaded from AnnoTree (90) was used. The bar chart shows the total number of available genomes (blue) and genomes containing functional PYP (red). Percentages of PYP-containing genomes are shown in red. Bacterial phyla with fewer than five PYP-containing genomes are not shown (*Gemmatimonadota*, *Actinobacteria*, *Acidobacteria*, *Armatimonadota*, *Thermoplasmatota*, and *Bdellovibrionota*).

The PAS superfamily is the largest group of sensory domains that are found in all three domains of life, bacteria, archaea, and eukarya (31), but the distribution of its structural prototype, PYP, has never been investigated. In our analysis, we found PYP homologs in 10 bacterial phyla (*Proteobacteria*, *Myxococcota*, *Spirochaetota*, *Bacteroidota*, *Gemmatimonadota*, *Actinobacteria*, *Acidobacteria*, *Armatimonadota*, *Thermoplasmatota*, and *Bdellovibrionota*), out of more than a hundred phyla defined by the Genome Taxonomy Database (69). In six of these phyla, fewer than five genomes contained PYP, indicating horizontal gene transfer. Thus, PYP has a narrow phyletic distribution (Fig. 5). Interestingly, the *Spirochaetota* and *Bacteroidota*, compared to the other two phyla, have relatively few functional PYPs (29 genomes and 26 genomes, respectively) (Fig. 5). The highest percentage of PYP-containing genomes is in the *Myxococcota* (Fig. 5). Furthermore, all three orders within this phylum have PYP-containing genomes, which is not observed for any other phyla (Fig. S4). Taken together, these observations suggest that PYP likely originated from the *Myxococcota* and that all instances of PYP occurrences in other bacterial phyla are the result of horizontal gene transfer.

## DISCUSSION

**FIG 6:**
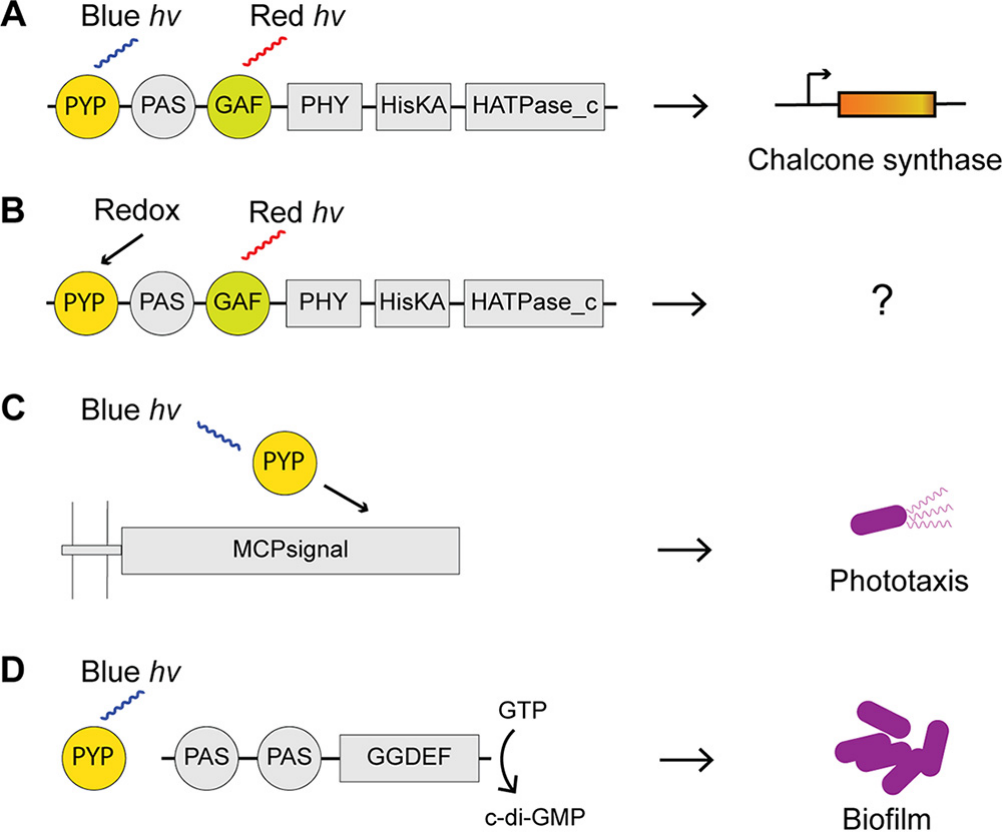
Schemes of signal transduction pathways initiated by the PYP sensor. Protein domain nomenclature is according to the Pfam database. (A) *R. centenum* Ppr is regulated by blue and red light and has a putative function in activating the chalcone synthase gene via a yet-to-be-identified response regulator. (B) Some methylobacterial Pprs have mutations in PYP that may lead to redox sensing. Their targets remain to be identified. (C) *H. halophila* and *Leptospira* PYPs may regulate phototaxis by binding to membrane-bound chemoreceptors that lack sensory domains. (D) PYPs from *I. loihiensis* and some other clade C homologs may modulate adjacent GGDEF/EAL proteins that control c-di-GMP levels and biofilm formation.

Our analysis suggests that a well-studied PYP protein from *H. halophila* is a member of a small, distinct, and evolutionarily novel family of PAS domains. We found that the PYP family is comprised of three distinct protein groups, one of which (clade C) is poorly characterized and might have novel functional properties. Domain architecture and gene neighborhood analyses revealed the functional diversity of PYP and suggested its involvement as a sensor in various signal transduction pathways controlling important cellular functions such as gene expression, motility, and biofilm formation (Fig. 6).

A previous study classified PYP homologs from 26 species into seven groups based on sequence similarity (43). Based on the present phylogenetic analyses using a much larger data set, we suggest keeping group 4 and group 6 as separate clades (clade B and clade C, respectively, in our analysis) while merging the remaining five groups into clade A. To date, hundreds of PYP genes have been discovered in various species (41, 43); however, fewer than 10 of them have been studied with respect to their structural and functional properties. Most of these studies were carried out using representatives of clade A, including the prototype *H. halophila* PYP (26–28), the Ppr sensor histidine kinase from *R. centenum* (40, 45, 54), a biofilm regulating c-di-GMP cyclase from Idiomarina loihiensis (42), and a UV-A sensor from Rhodobacter capsulatus (46, 65). Although the PYP domains from all of these proteins are located in the same clade, differences in their recovery kinetics (40), functional outputs (42), and light activation (46) have been reported. More recently, studies on PYPs from L. biflexa (44) and S. ruber (47), which belong to clade B, have revealed novel photocycles. As for clade C, several PYPs from the *Burkholderiales* were reported (41, 43), but none of their properties have been thoroughly characterized.

From our data set, we identified three common mutations in PYP at the pCA-binding site: Y42F, E46Q, and various substitutions of Cys69. Most interestingly, although a single mutation of either Tyr42 or Glu46 can still retain pCA binding and protein function, we never found the two mutations occurring in the same functional PYP (i.e., with Cys69). This suggests that besides Cys69, at least one of the other two residues is required for hydrogen bond formation and retaining the photocycle. A study on *S. ruber* showed that in the PYP Y42F/E46Q double mutant, pCA always prefers the protonated state (70), which may cause photocycle initiation to fail.

Other than Tyr42 and Glu46, we found substitutions of Cys69 in proteins from clades A, B, and the PYP_like branch. Many of these mutants are found in Ppr homologs from methylobacteria. Although the function of methylobacterial Ppr is unknown, the closely related *R. centenum* Ppr was proposed to regulate chalcone synthase, which is involved in the biosynthesis of photoprotective flavonoids (54). Methylobacteria are phytosymbionts living on plants (71), where UV is a common threat in the environment. Intriguingly, methylobacteria are known to produce UV-absorbing compounds, including carotenoids and methylobamine (71–73), which may serve as photoprotectors. With the substitution at Cys69, a group of Ppr-PYPs in methylobacteria evolved two novel cysteines via Y42C and S50C mutations (Fig. 3B; see also Fig. S3 in the supplemental material), suggesting a conversion of the Ppr biological function from light sensing (Fig. 6A) to redox sensing (Fig. 6B).

Based on the gene neighborhood, we identified various potential signal transduction pathways for PYP. For example, we found that the *Leptospira* PYPs in clade B are always located adjacent to chemoreceptors (Fig. 4B; Data Set S3). These chemoreceptors have transmembrane regions followed by signaling modules but lack sensory domains. This architecture resembles a that of set of chemotaxis proteins in Campylobacter jejuni: CetB is a PAS domain protein that contains flavin adenine dinucleotide (FAD) as a cofactor and detects redox states from the electron transport chain in the cell membrane, and CetA is a chemoreceptor signaling domain that receives signals from CetB and transduces signals to the downstream CheA kinase (74, 75). Similarly, in *Leptospira*, PYP might play the role of a sensor that detects the signal and relays it to the chemoreceptor encoded adjacent to PYP (Fig. 6C). We found many instances of GGDEF and EAL (c-di-GMP phosphodiesterase) domain proteins encoded in the gene neighborhood of PYPs from clade C (Fig. 4B; Data Set S3), similar to the signal transduction system in I. loihiensis from clade A (Fig. 4B), where PYP putatively binds the adjacent GGDEF protein to regulate biofilm formation by controlling the intracellular levels of c-di-GMP (42) (Fig. 6D).

Finally, we showed that PYP has a narrow taxonomic distribution, suggesting its evolutionary novelty. Two scenarios for the origin and evolution of PYP are conceivable. (i) PYP evolved from the common ancestor of the *Proteobacteria* and *Myxococcota*. While many *Myxococcota* species from all three orders maintain PYP genes, half of the *Proteobacteria* orders underwent gene loss. (ii) PYP evolved within the *Myxococcota* and was later horizontally transferred to closely related members of the *Proteobacteria* and a few other more distantly related bacterial phyla. Under either scenario, PYP likely evolved from a generic PAS domain by acquiring a key residue, Cys69, for its chromophore binding and further optimizing its binding pocket to become a successful light sensor.

## MATERIALS AND METHODS

### Data sources and bioinformatics software.

BLAST (50) searches were carried out against the NCBI Reference Sequence (RefSeq) protein database (51) (July 2021) using full-length PYP sequences from various bacterial phyla (NCBI accession numbers WP_011814604.1 for *H. halophila* PYP, WP_075010582.1 for *Myxococcota*, WP_002975717.1 for *Spirochaetota*, WP_011404950.1 for *Bacteroidota*, WP_026849937.1 for *Gemmatimonadota*, WP_091439124.1 for *Actinobacteria*, and WP_052571869.1 for *Acidobacteria*) as queries, with an E value threshold of ≤0.01. Taxonomy information was retrieved from the Genome Taxonomy Database, release 202 (69). Domain architectures and gene neighborhoods were identified using HMMER (52), HHpred (76), CDvist (77), and TREND (78, 79) using default parameters (E value of ≤0.01 for HMMER, probability of ≥20% for HHpred, and probability of ≥60% for CDvist). Multiple-sequence alignments were constructed using the MAFFT v7 L-INS-i algorithm (80). Alignments were edited in Jalview v2.11.0 (81). Sequence logos were generated using WebLogo 3 (82). The maximum likelihood tree was built in MEGA-X (83) using the Jones-Taylor-Thornton (JTT) substitution model and edited in iTOL v6.5.7 (84). Protein structures were visualized in PyMOL v2.5.0 (85). Networks were constructed using Cytoscape v3.8.2 (86). Protein structures were predicted using AlphaFold (87). Ligand-binding simulations were conducted using AutoDock Vina (88).

### Clustering protein sequences using the Markov cluster algorithm.

A total of 50,834 full-length sequences of the PAS family (Pfam entry PF00989; InterPro entry IPR013767) were retrieved from Pfam 33.1 (49). Sequence redundancy was reduced at the 95% identity level using CD-HIT (89). An all-versus-all BLAST analysis was conducted using ncbi-blast-2.10.0+ (50). BLAST hits were filtered with a coverage threshold of ≥80% and an E value threshold of ≤0.05 using a custom python script. The unweighted network, constructed based on the filtered hits, was used to identify clusters by running the Markov cluster algorithm with an inflation value of 1.4 (48). The size of each cluster node is proportional to the logarithm of the number of sequences within each cluster to base 10. The weights of edges connecting clusters in the network were calculated as the number of all of the observed mutual BLAST hits between the two clusters divided by the number of all possible mutual BLAST hits between the two clusters.

### Building a profile hidden Markov model of the PYP family.

The multiple-sequence alignment was built using the L-INS-i algorithm of the MAFFT package (80). Sequence redundancy was reduced at 80% identities using Jalview (81). The hidden Markov model (HMM) from the resulting sequences was built using the hmmbuild command of the HMMER package (52). Sequences matching the HMM were obtained from the UniProt Reference Proteomes database using the hmmsearch command (52). Finally, the above-described steps were repeated, resulting in the final HMM. Noise, trusted, and gathering cutoffs of the model were set based on the conservation of the Cys69 residue.

## References

[1] van der Horst MA, Hellingwerf KJ. 2004. Photoreceptor proteins, “star actors of modern times”: a review of the functional dynamics in the structure of representative members of six different photoreceptor families. Acc Chem Res 37:13–20. 10.1021/ar020219d. 14730990

[2] Liscum E, Askinosie SK, Leuchtman DL, Morrow J, Willenburg KT, Coats DR. 2014. Phototropism: growing towards an understanding of plant movement. Plant Cell 26:38–55. 10.1105/tpc.113.119727. 24481074 PMC3963583

[3] Lamers J, van der Meer T, Testerink C. 2020. How plants sense and respond to stressful environments. Plant Physiol 182:1624–1635. 10.1104/pp.19.01464. 32132112 PMC7140927

[4] Chaves I, Pokorny R, Byrdin M, Hoang N, Ritz T, Brettel K, Essen LO, van der Horst GT, Batschauer A, Ahmad M. 2011. The cryptochromes: blue light photoreceptors in plants and animals. Annu Rev Plant Biol 62:335–364. 10.1146/annurev-arplant-042110-103759. 21526969

[5] Paik I, Huq E. 2019. Plant photoreceptors: multi-functional sensory proteins and their signaling networks. Semin Cell Dev Biol 92:114–121. 10.1016/j.semcdb.2019.03.007. 30946988 PMC8630751

[6] Mroginski M-A, Adam S, Amoyal GS, Barnoy A, Bondar A-N, Borin VA, Church JR, Domratcheva T, Ensing B, Fanelli F, Ferre N, Filiba O, Pedraza-Gonzalez L, Gonzalez R, Gonzalez-Espinoza CE, Kar RK, Kemmler L, Kim SS, Kongsted J, Krylov AI, Lahav Y, Lazaratos M, NasserEddin Q, Navizet I, Nemukhin A, Olivucci M, Olsen JMH, Perez de Alba Ortiz A, Pieri E, Rao AG, Rhee YM, Ricardi N, Sen S, Solov’yov IA, De Vico L, Wesolowski TA, Wiebeler C, Yang X, Schapiro I. 2021. Frontiers in multiscale modeling of photoreceptor proteins. Photochem Photobiol 97:243–269. 10.1111/php.13372. 33369749 PMC9185909

[7] Chudakov DM, Matz MV, Lukyanov S, Lukyanov KA. 2010. Fluorescent proteins and their applications in imaging living cells and tissues. Physiol Rev 90:1103–1163. 10.1152/physrev.00038.2009. 20664080

[8] Meyer TE. 1985. Isolation and characterization of soluble cytochromes, ferredoxins and other chromophoric proteins from the halophilic phototrophic bacterium Ectothiorhodospira halophila. Biochim Biophys Acta 806:175–183. 10.1016/0005-2728(85)90094-5. 2981543

[9] Kumar N, Hori Y, Kikuchi K. 2019. Photoactive yellow protein and its chemical probes: an approach to protein labelling in living cells. J Biochem 166:121–127. 10.1093/jb/mvz051. 31340005

[10] Seong J, Lin MZ. 2021. Optobiochemistry: genetically encoded control of protein activity by light. Annu Rev Biochem 90:475–501. 10.1146/annurev-biochem-072420-112431. 33781076 PMC11464261

[11] Plamont M-A, Billon-Denis E, Maurin S, Gauron C, Pimenta FM, Specht CG, Shi J, Querard J, Pan BY, Rossignol J, Moncoq K, Morellet N, Volovitch M, Lescop E, Chen Y, Triller A, Vriz S, Le Saux T, Jullien L, Gautier A. 2016. Correction for Plamont et al., Small fluorescence-activating and absorption-shifting tag for tunable protein imaging in vivo. Proc Natl Acad Sci USA 113:E1412. 10.1073/pnas.1602162113. 26711992 PMC4725535

[12] Hori Y, Otomura N, Nishida A, Nishiura M, Umeno M, Suetake I, Kikuchi K. 2018. Synthetic-molecule/protein hybrid probe with fluorogenic switch for live-cell imaging of DNA methylation. J Am Chem Soc 140:1686–1690. 10.1021/jacs.7b09713. 29381073

[13] Ali AM, Reis JM, Xia Y, Rashid AJ, Mercaldo V, Walters BJ, Brechun KE, Borisenko V, Josselyn SA, Karanicolas J, Woolley GA. 2015. Optogenetic inhibitor of the transcription factor CREB. Chem Biol 22:1531–1539. 10.1016/j.chembiol.2015.09.018. 26590638 PMC4656143

[14] Sprenger WW, Hoff WD, Armitage JP, Hellingwerf KJ. 1993. The eubacterium Ectothiorhodospira halophila is negatively phototactic, with a wavelength dependence that fits the absorption spectrum of the photoactive yellow protein. J Bacteriol 175:3096–3104. 10.1128/jb.175.10.3096-3104.1993. 8491725 PMC204631

[15] Van Beeumen JJ, Devreese BV, Van Bun SM, Hoff WD, Hellingwerf KJ, Meyer TE, McRee DE, Cusanovich MA. 1993. Primary structure of a photoactive yellow protein from the phototrophic bacterium Ectothiorhodospira halophila, with evidence for the mass and the binding site of the chromophore. Protein Sci 2:1114–1125. 10.1002/pro.5560020706. 8358295 PMC2142427

[16] Hoff WD, Düx P, Hård K, Devreese B, Nugteren-Roodzant IM, Crielaard W, Boelens R, Kaptein R, van Beeumen J, Hellingwerf KJ. 1994. Thiol ester-linked p-coumaric acid as a new photoactive prosthetic group in a protein with rhodopsin-like photochemistry. Biochemistry 33:13959–13962. 10.1021/bi00251a001. 7947803

[17] Getzoff ED, Gutwin KN, Genick UK. 2003. Anticipatory active-site motions and chromophore distortion prime photoreceptor PYP for light activation. Nat Struct Biol 10:663–668. 10.1038/nsb958. 12872160

[18] Pande K, Hutchison CDM, Groenhof G, Aquila A, Robinson JS, Tenboer J, Basu S, Boutet S, DePonte DP, Liang MN, White TA, Zatsepin NA, Yefanov O, Morozov D, Oberthuer D, Gati C, Subramanian G, James D, Zhao Y, Koralek J, Brayshaw J, Kupitz C, Conrad C, Roy-Chowdhury S, Coe JD, Metz M, Xavier PL, Grant TD, Koglin JE, Ketawala G, Fromme R, Srajer V, Henning R, Spence JCH, Ourmazd A, Schwander P, Weierstall U, Frank M, Fromme P, Barty A, Chapman HN, Moffat K, van Thor JJ, Schmidt M. 2016. Femtosecond structural dynamics drives the trans/cis isomerization in photoactive yellow protein. Science 352:725–729. 10.1126/science.aad5081. 27151871 PMC5291079

[19] Hoff WD, van Stokkum IH, van Ramesdonk HJ, van Brederode ME, Brouwer AM, Fitch JC, Meyer TE, van Grondelle R, Hellingwerf KJ. 1994. Measurement and global analysis of the absorbance changes in the photocycle of the photoactive yellow protein from Ectothiorhodospira halophila. Biophys J 67:1691–1705. 10.1016/S0006-3495(94)80643-5. 7819501 PMC1225531

[20] Imamoto Y, Mihara K, Hisatomi O, Kataoka M, Tokunaga F, Bojkova N, Yoshihara K. 1997. Evidence for proton transfer from Glu-46 to the chromophore during the photocycle of photoactive yellow protein. J Biol Chem 272:12905–12908. 10.1074/jbc.272.20.12905. 9148894

[21] Pan DH, Philip A, Hoff WD, Mathies RA. 2004. Time-resolved resonance Raman structural studies of the pB′ intermediate in the photocycle of photoactive yellow protein. Biophys J 86:2374–2382. 10.1016/S0006-3495(04)74294-0. 15041675 PMC1304086

[22] Xie AH, Kelemen L, Hendriks J, White BJ, Hellingwerf KJ, Hoff WD. 2001. Formation of a new buried charge drives a large-amplitude protein quake in photoreceptor activation. Biochemistry 40:1510–1517. 10.1021/bi002449a. 11327809

[23] Ramachandran PL, Lovett JE, Carl PJ, Cammarata M, Lee JH, Jung YO, Ihee H, Timmel CR, van Thor JJ. 2011. The short-lived signaling state of the photoactive yellow protein photoreceptor revealed by combined structural probes. J Am Chem Soc 133:9395–9404. 10.1021/ja200617t. 21627157

[24] Hendriks J, van Stokkum IHM, Crielaard W, Hellingwerf KJ. 1999. Kinetics of and intermediates in a photocycle branching reaction of the photoactive yellow protein from Ectothiorhodospira halophila. FEBS Lett 458:252–256. 10.1016/S0014-5793(99)01136-9. 10481075

[25] Carroll EC, Song SH, Kumauchi M, van Stokkum IHM, Jailaubekov A, Hoff WD, Larsen DS. 2010. Subpicosecond excited-state proton transfer preceding isomerization during the photorecovery of photoactive yellow protein. J Phys Chem Lett 1:2793–2799. 10.1021/jz101049v. 20953237 PMC2955422

[26] Pellequer JL, Wager-Smith KA, Kay SA, Getzoff ED. 1998. Photoactive yellow protein: a structural prototype for the three-dimensional fold of the PAS domain superfamily. Proc Natl Acad Sci USA 95:5884–5890. 10.1073/pnas.95.11.5884. 9600888 PMC34491

[27] Cusanovich MA, Meyer TE. 2003. Photoactive yellow protein: a prototypic PAS domain sensory protein and development of a common signaling mechanism. Biochemistry 42:4759–4770. 10.1021/bi020690e. 12718516

[28] Imamoto Y, Kataoka M. 2007. Structure and photoreaction of photoactive yellow protein, a structural prototype of the PAS domain superfamily. Photochem Photobiol 83:40–49. 10.1562/2006-02-28-IR-827. 16939366

[29] Vreede J, van der Horst MA, Hellingwerf KJ, Crielaard W, van Aalten DM. 2003. PAS domains. Common structure and common flexibility. J Biol Chem 278:18434–18439. 10.1074/jbc.M301701200. 12639952

[30] Moglich A, Ayers RA, Moffat K. 2009. Structure and signaling mechanism of Per-ARNT-Sim domains. Structure 17:1282–1294. 10.1016/j.str.2009.08.011. 19836329 PMC3092527

[31] Taylor BL, Zhulin IB. 1999. PAS domains: internal sensors of oxygen, redox potential, and light. Microbiol Mol Biol Rev 63:479–506. 10.1128/MMBR.63.2.479-506.1999. 10357859 PMC98974

[32] Henry JT, Crosson S. 2011. Ligand-binding PAS domains in a genomic, cellular, and structural context. Annu Rev Microbiol 65:261–286. 10.1146/annurev-micro-121809-151631. 21663441 PMC3298442

[33] Stuffle EC, Johnson MS, Watts KJ. 2021. PAS domains in bacterial signal transduction. Curr Opin Microbiol 61:8–15. 10.1016/j.mib.2021.01.004. 33647528 PMC8169565

[34] Edwards HE, Gorelick DA. 2022. The evolution and structure/function of bHLH-PAS transcription factor family. Biochem Soc Trans 50:1227–1243. 10.1042/BST20211225. 35695677 PMC10584024

[35] Anstoter CS, Curchod BFE, Verlet JRR. 2020. Geometric and electronic structure probed along the isomerisation coordinate of a photoactive yellow protein chromophore. Nat Commun 11:2827. 10.1038/s41467-020-16667-x. 32499507 PMC7272410

[36] Konold PE, Arik E, Weißenborn J, Arents JC, Hellingwerf KJ, van Stokkum IHM, Kennis JTM, Groot ML. 2020. Confinement in crystal lattice alters entire photocycle pathway of the photoactive yellow protein. Nat Commun 11:4248. 10.1038/s41467-020-18065-9. 32843623 PMC7447820

[37] Pandey S, Bean R, Sato T, Poudyal I, Bielecki J, Cruz Villarreal J, Yefanov O, Mariani V, White TA, Kupitz C, Hunter M, Abdellatif MH, Bajt S, Bondar V, Echelmeier A, Doppler D, Emons M, Frank M, Fromme R, Gevorkov Y, Giovanetti G, Jiang M, Kim D, Kim Y, Kirkwood H, Klimovskaia A, Knoska J, Koua FHM, Letrun R, Lisova S, Maia L, Mazalova V, Meza D, Michelat T, Ourmazd A, Palmer G, Ramilli M, Schubert R, Schwander P, Silenzi A, Sztuk-Dambietz J, Tolstikova A, Chapman HN, Ros A, Barty A, Fromme P, Mancuso AP, Schmidt M. 2020. Time-resolved serial femtosecond crystallography at the European XFEL. Nat Methods 17:73–78. 10.1038/s41592-019-0628-z. 31740816 PMC9113060

[38] Hosseinizadeh A, Breckwoldt N, Fung R, Sepehr R, Schmidt M, Schwander P, Santra R, Ourmazd A. 2021. Few-fs resolution of a photoactive protein traversing a conical intersection. Nature 599:697–701. 10.1038/s41586-021-04050-9. 34732893

[39] Schmidt M. 2017. A short history of structure based research on the photocycle of photoactive yellow protein. Struct Dyn 4:e032201. 10.1063/1.4974172. PMC5291790 28191482

[40] Rajagopal S, Moffat K. 2003. Crystal structure of a photoactive yellow protein from a sensor histidine kinase: conformational variability and signal transduction. Proc Natl Acad Sci USA 100:1649–1654. 10.1073/pnas.0336353100. 12563032 PMC149887

[41] Kumauchi M, Hara MT, Stalcup P, Xie A, Hoff WD. 2008. Identification of six new photoactive yellow proteins—diversity and structure-function relationships in a bacterial blue light photoreceptor. Photochem Photobiol 84:956–969. 10.1111/j.1751-1097.2008.00335.x. 18399917

[42] van der Horst MA, Stalcup TP, Kaledhonkar S, Kumauchi M, Hara M, Xie A, Hellingwerf KJ, Hoff WD. 2009. Locked chromophore analogs reveal that photoactive yellow protein regulates biofilm formation in the deep sea bacterium Idiomarina loihiensis. J Am Chem Soc 131:17443–17451. 10.1021/ja9057103. 19891493

[43] Meyer TE, Kyndt JA, Memmi S, Moser T, Colon-Acevedo B, Devreese B, Van Beeumen JJ. 2012. The growing family of photoactive yellow proteins and their presumed functional roles. Photochem Photobiol Sci 11:1495–1514. 10.1039/c2pp25090j. 22911088

[44] Mix LT, Kirpich J, Kumauchi M, Ren J, Vengris M, Hoff WD, Larsen DS. 2016. Bifurcation in the ultrafast dynamics of the photoactive yellow proteins from Leptospira biflexa and Halorhodospira halophila. Biochemistry 55:6138–6149. 10.1021/acs.biochem.6b00547. 27749038

[45] Mix LT, Hara M, Rathod R, Kumauchi M, Hoff WD, Larsen DS. 2016. Noncanonical photocycle initiation dynamics of the photoactive yellow protein (PYP) domain of the PYP-phytochrome-related (Ppr) photoreceptor. J Phys Chem Lett 7:5212–5218. 10.1021/acs.jpclett.6b02253. 27973895

[46] Kim S, Nakasone Y, Takakado A, Yamazaki Y, Kamikubo H, Terazima M. 2021. A unique photochromic UV-A sensor protein, Rc-PYP, interacting with the PYP-binding protein. Phys Chem Chem Phys 23:17813–17825. 10.1039/d1cp02731j. 34397052

[47] Mix LT, Hara M, Fuzell J, Kumauchi M, Kaledhonkar S, Xie AH, Hoff WD, Larsen DS. 2021. Not all photoactive yellow proteins are built alike: surprises and insights into chromophore photoisomerization, protonation, and thermal reisomerization of the photoactive yellow protein isolated from Salinibacter ruber. J Am Chem Soc 143:19614–19628. 10.1021/jacs.1c08910. 34780163

[48] Schaeffer SE. 2007. Graph clustering. Comput Sci Rev 1:27–64. 10.1016/j.cosrev.2007.05.001.

[49] Mistry J, Chuguransky S, Williams L, Qureshi M, Salazar GA, Sonnhammer ELL, Tosatto SCE, Paladin L, Raj S, Richardson LJ, Finn RD, Bateman A. 2021. Pfam: the protein families database in 2021. Nucleic Acids Res 49:D412–D419. 10.1093/nar/gkaa913. 33125078 PMC7779014

[50] Altschul SF, Madden TL, Schaffer AA, Zhang J, Zhang Z, Miller W, Lipman DJ. 1997. Gapped BLAST and PSI-BLAST: a new generation of protein database search programs. Nucleic Acids Res 25:3389–3402. 10.1093/nar/25.17.3389. 9254694 PMC146917

[51] O’Leary NA, Wright MW, Brister JR, Ciufo S, Haddad D, McVeigh R, Rajput B, Robbertse B, Smith-White B, Ako-Adjei D, Astashyn A, Badretdin A, Bao Y, Blinkova O, Brover V, Chetvernin V, Choi J, Cox E, Ermolaeva O, Farrell CM, Goldfarb T, Gupta T, Haft D, Hatcher E, Hlavina W, Joardar VS, Kodali VK, Li W, Maglott D, Masterson P, McGarvey KM, Murphy MR, O’Neill K, Pujar S, Rangwala SH, Rausch D, Riddick LD, Schoch C, Shkeda A, Storz SS, Sun H, Thibaud-Nissen F, Tolstoy I, Tully RE, Vatsan AR, Wallin C, Webb D, Wu W, Landrum MJ, Kimchi A, et al. 2016. Reference Sequence (RefSeq) database at NCBI: current status, taxonomic expansion, and functional annotation. Nucleic Acids Res 44:D733–D745. 10.1093/nar/gkv1189. 26553804 PMC4702849

[52] Eddy SR. 2011. Accelerated profile HMM searches. PLoS Comput Biol 7:e1002195. 10.1371/journal.pcbi.1002195. 22039361 PMC3197634

[53] Haft DH, Selengut JD, Richter RA, Harkins D, Basu MK, Beck E. 2013. TIGRFAMs and genome properties in 2013. Nucleic Acids Res 41:D387–D395. 10.1093/nar/gks1234. 23197656 PMC3531188

[54] Jiang Z, Swem LR, Rushing BG, Devanathan S, Tollin G, Bauer CE. 1999. Bacterial photoreceptor with similarity to photoactive yellow protein and plant phytochromes. Science 285:406–409. 10.1126/science.285.5426.406. 10411503

[55] Morishita T, Harigai M, Yamazaki Y, Kamikubo H, Kataoka M, Imamoto Y. 2007. Array of aromatic amino acid side chains located near the chromophore of photoactive yellow protein. Photochem Photobiol 83:280–285. 10.1562/2006-06-15-RA-929. 16879039

[56] Kyndt JA, Fitch JC, Meyer TE, Cusanovich MA. 2005. Thermochromatium tepidum photoactive yellow protein/bacteriophytochrome/diguanylate cyclase: characterization of the PYP domain. Biochemistry 44:4755–4764. 10.1021/bi047373n. 15779902

[57] Carroll EC, Hospes M, Valladares C, Hellingwerf KJ, Larsen DS. 2011. Is the photoactive yellow protein a UV-B/blue light photoreceptor? Photochem Photobiol Sci 10:464–468. 10.1039/c0pp00274g. 21267495

[58] van der Horst MA, Arents JC, Kort R, Hellingwerf KJ. 2007. Binding, tuning and mechanical function of the 4-hydroxy-cinnamic acid chromophore in photoactive yellow protein. Photochem Photobiol Sci 6:571–579. 10.1039/b701072a. 17487311

[59] Philip AF, Kumauchi M, Hoff WD. 2010. Robustness and evolvability in the functional anatomy of a PER-ARNT-SIM (PAS) domain. Proc Natl Acad Sci USA 107:17986–17991. 10.1073/pnas.1004823107. 20889915 PMC2964196

[60] Imamoto Y, Koshimizu H, Mihara K, Hisatomi O, Mizukami T, Tsujimoto K, Kataoka M, Tokunaga F. 2001. Roles of amino acid residues near the chromophore of photoactive yellow protein. Biochemistry 40:4679–4685. 10.1021/bi002291u. 11294635

[61] Sugishima M, Tanimoto N, Soda K, Hamada N, Tokunaga F, Fukuyama K. 2004. Structure of photoactive yellow protein (PYP) E46Q mutant at 1.2 A resolution suggests how Glu46 controls the spectroscopic and kinetic characteristics of PYP. Acta Crystallogr D Biol Crystallogr 60:2305–2309. 10.1107/S0907444904024084. 15583378

[62] Genick UK, Devanathan S, Meyer TE, Canestrelli IL, Williams E, Cusanovich MA, Tollin G, Getzoff ED. 1997. Active site mutants implicate key residues for control of color and light cycle kinetics of photoactive yellow protein. Biochemistry 36:8–14. 10.1021/bi9622884. 8993312

[63] Yang C, Kim TW, Kim Y, Choi J, Lee SJ, Ihee H. 2017. Kinetics of the E46Q mutant of photoactive yellow protein investigated by transient grating spectroscopy. Chem Phys Lett 683:262–267. 10.1016/j.cplett.2017.03.018.

[64] Brudler R, Meyer TE, Genick UK, Devanathan S, Woo TT, Millar DP, Gerwert K, Cusanovich MA, Tollin G, Getzoff ED. 2000. Coupling of hydrogen bonding to chromophore conformation and function in photoactive yellow protein. Biochemistry 39:13478–13486. 10.1021/bi0009946. 11063584

[65] Kyndt JA, Hurley JK, Devreese B, Meyer TE, Cusanovich MA, Tollin G, Van Beeumen JJ. 2004. Rhodobacter capsulatus photoactive yellow protein: genetic context, spectral and kinetics characterization, and mutagenesis. Biochemistry 43:1809–1820. 10.1021/bi035789f. 14967022

[66] Kyndt JA, Vanrobaeys F, Fitch JC, Devreese BV, Meyer TE, Cusanovich MA, Van Beeumen JJ. 2003. Heterologous production of Halorhodospira halophila holo-photoactive yellow protein through tandem expression of the postulated biosynthetic genes. Biochemistry 42:965–970. 10.1021/bi027037b. 12549916

[67] Kyndt JA, Meyer TE, Cusanovich MA, Van Beeumen JJ. 2002. Characterization of a bacterial tyrosine ammonia lyase, a biosynthetic enzyme for the photoactive yellow protein. FEBS Lett 512:240–244. 10.1016/S0014-5793(02)02272-X. 11852088

[68] Koike M, Reed LJ, Carroll WR. 1963. α-Keto acid dehydrogenation complexes. IV. Resolution and reconstitution of the Escherichia coli pyruvate dehydrogenation complex. J Biol Chem 238:30–39. 10.1016/S0021-9258(19)83957-1. 14034257

[69] Parks DH, Chuvochina M, Waite DW, Rinke C, Skarshewski A, Chaumeil PA, Hugenholtz P. 2018. A standardized bacterial taxonomy based on genome phylogeny substantially revises the tree of life. Nat Biotechnol 36:996–1004. 10.1038/nbt.4229. 30148503

[70] Lin C-Y, Boxer SG. 2020. Unusual spectroscopic and electric field sensitivity of chromophores with short hydrogen bonds: GFP and PYP as model systems. J Phys Chem B 124:9513–9525. 10.1021/acs.jpcb.0c07730. 33073990 PMC8549526

[71] Kutschera U. 2007. Plant-associated methylobacteria as co-evolved phytosymbionts: a hypothesis. Plant Signal Behav 2:74–78. 10.4161/psb.2.2.4073. 19516971 PMC2633902

[72] Yoshida S, Hiradate S, Koitabashi M, Kamo T, Tsushima S. 2017. Phyllosphere Methylobacterium bacteria contain UVA-absorbing compounds. J Photochem Photobiol B 167:168–175. 10.1016/j.jphotobiol.2016.12.019. 28068611

[73] Kamo T, Hiradate S, Suzuki K, Fujita I, Yamaki S, Yoneda T, Koitabashi M, Yoshida S. 2018. Methylobamine, a UVA-absorbing compound from the plant-associated bacteria Methylobacterium sp. Nat Prod Commun 13:141–143.

[74] Taylor BL, Zhulin IB, Johnson MS. 1999. Aerotaxis and other energy-sensing behavior in bacteria. Annu Rev Microbiol 53:103–128. 10.1146/annurev.micro.53.1.103. 10547687

[75] Elliott KT, Dirita VJ. 2008. Characterization of CetA and CetB, a bipartite energy taxis system in Campylobacter jejuni. Mol Microbiol 69:1091–1103. 10.1111/j.1365-2958.2008.06357.x. 18631239 PMC2628428

[76] Zimmermann L, Stephens A, Nam SZ, Rau D, Kubler J, Lozajic M, Gabler F, Soding J, Lupas AN, Alva V. 2018. A completely reimplemented MPI bioinformatics toolkit with a new HHpred server at its core. J Mol Biol 430:2237–2243. 10.1016/j.jmb.2017.12.007. 29258817

[77] Adebali O, Ortega DR, Zhulin IB. 2015. CDvist: a webserver for identification and visualization of conserved domains in protein sequences. Bioinformatics 31:1475–1477. 10.1093/bioinformatics/btu836. 25527097 PMC4410658

[78] Gumerov VM, Zhulin IB. 2020. TREND: a platform for exploring protein function in prokaryotes based on phylogenetic, domain architecture and gene neighborhood analyses. Nucleic Acids Res 48:W72–W76. 10.1093/nar/gkaa243. 32282909 PMC7319448

[79] Gumerov VM, Zhulin IB. 2022. Correction to ‘TREND: a platform for exploring protein function in prokaryotes based on phylogenetic, domain architecture and gene neighborhood analyses’. Nucleic Acids Res 50:1795. 10.1093/nar/gkac034. 35100406 PMC8860576

[80] Katoh K, Rozewicki J, Yamada KD. 2019. MAFFT online service: multiple sequence alignment, interactive sequence choice and visualization. Brief Bioinform 20:1160–1166. 10.1093/bib/bbx108. 28968734 PMC6781576

[81] Waterhouse AM, Procter JB, Martin DM, Clamp M, Barton GJ. 2009. Jalview version 2—a multiple sequence alignment editor and analysis workbench. Bioinformatics 25:1189–1191. 10.1093/bioinformatics/btp033. 19151095 PMC2672624

[82] Crooks GE, Hon G, Chandonia JM, Brenner SE. 2004. WebLogo: a sequence logo generator. Genome Res 14:1188–1190. 10.1101/gr.849004. 15173120 PMC419797

[83] Kumar S, Stecher G, Li M, Knyaz C, Tamura K. 2018. MEGA X: molecular evolutionary genetics analysis across computing platforms. Mol Biol Evol 35:1547–1549. 10.1093/molbev/msy096. 29722887 PMC5967553

[84] Letunic I, Bork P. 2021. Interactive Tree of Life (iTOL) v5: an online tool for phylogenetic tree display and annotation. Nucleic Acids Res 49:W293–W296. 10.1093/nar/gkab301. 33885785 PMC8265157

[85] Schrödinger, LLC. 2015. The PyMOL molecular graphics system, version 2.0. Schrödinger, LLC, New York, NY.

[86] Shannon P, Markiel A, Ozier O, Baliga NS, Wang JT, Ramage D, Amin N, Schwikowski B, Ideker T. 2003. Cytoscape: a software environment for integrated models of biomolecular interaction networks. Genome Res 13:2498–2504. 10.1101/gr.1239303. 14597658 PMC403769

[87] Jumper J, Evans R, Pritzel A, Green T, Figurnov M, Ronneberger O, Tunyasuvunakool K, Bates R, Zidek A, Potapenko A, Bridgland A, Meyer C, Kohl SAA, Ballard AJ, Cowie A, Romera-Paredes B, Nikolov S, Jain R, Adler J, Back T, Petersen S, Reiman D, Clancy E, Zielinski M, Steinegger M, Pacholska M, Berghammer T, Bodenstein S, Silver D, Vinyals O, Senior AW, Kavukcuoglu K, Kohli P, Hassabis D. 2021. Highly accurate protein structure prediction with AlphaFold. Nature 596:583–589. 10.1038/s41586-021-03819-2. 34265844 PMC8371605

[88] Eberhardt J, Santos-Martins D, Tillack AF, Forli S. 2021. AutoDock Vina 1.2.0: new docking methods, expanded force field, and Python bindings. J Chem Inf Model 61:3891–3898. 10.1021/acs.jcim.1c00203. 34278794 PMC10683950

[89] Fu L, Niu B, Zhu Z, Wu S, Li W. 2012. CD-HIT: accelerated for clustering the next-generation sequencing data. Bioinformatics 28:3150–3152. 10.1093/bioinformatics/bts565. 23060610 PMC3516142

[90] Mendler K, Chen H, Parks DH, Lobb B, Hug LA, Doxey AC. 2019. AnnoTree: visualization and exploration of a functionally annotated microbial tree of life. Nucleic Acids Res 47:4442–4448. 10.1093/nar/gkz246. 31081040 PMC6511854

