# Supplementary material for: Photoactive Yellow Protein Represents a Distinct, Evolutionarily Novel Family of PAS Domains
Source: J Bacteriol. 2022 Oct 5;204(11):e00300-22. doi: 10.1128/jb.00300-22 (PMC9664959; doi:10.1128/jb.00300-22)
Supplement: Supplemental file 6 — Fig. S1 to S4 and Tables S1 to S3. Download jb.00300-22-s0001.pdf, PDF file, 1.2 MB [file jb.00300-22-s0001.pdf]

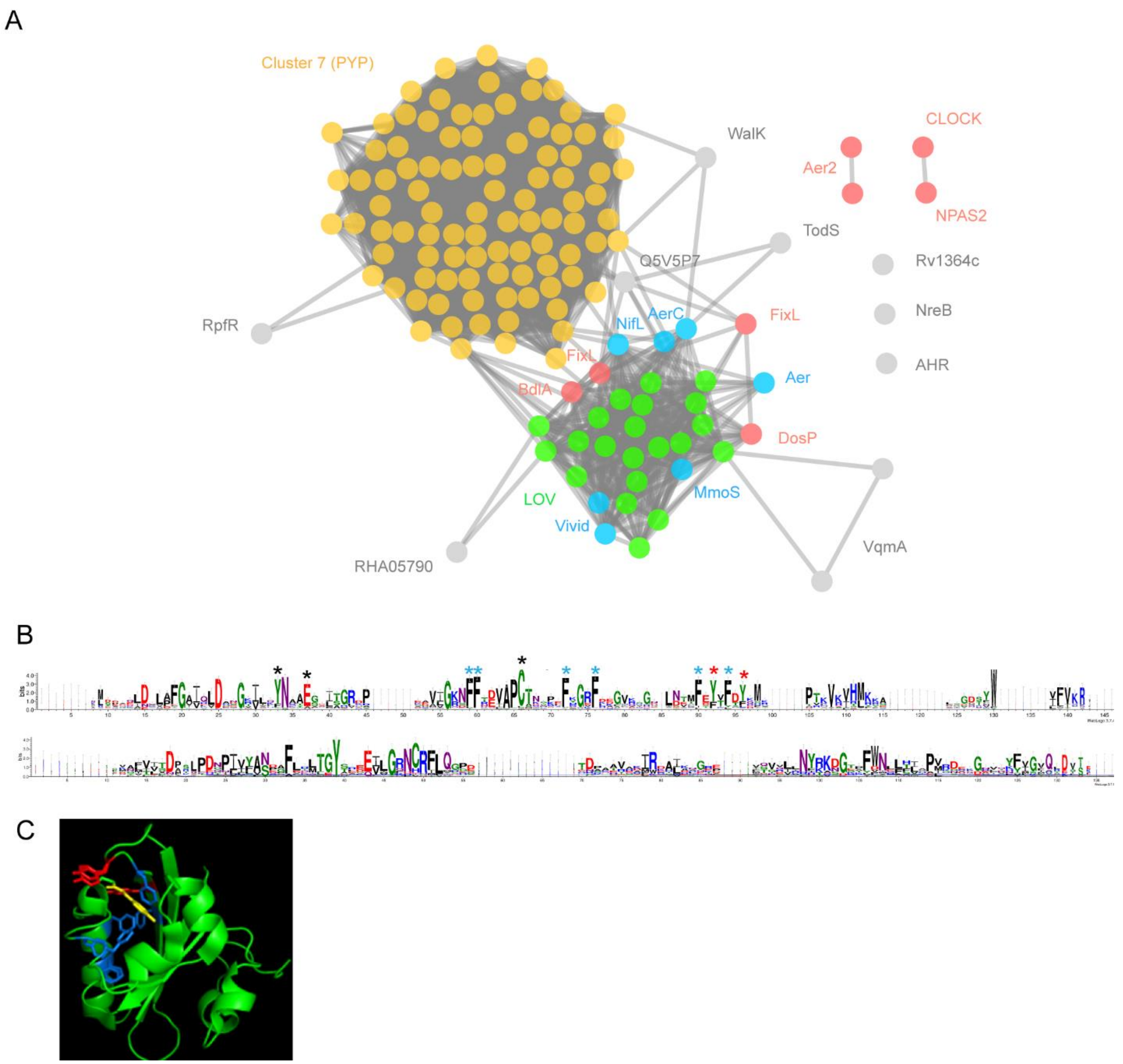

Fig. S1. **A.** Sequence similarity network of PAS domains with known ligands/cofactors (see Table S3). Each node represents a PAS domain sequence from the labelled protein. Nodes are connected if BLAST hits were found between them (E value <= 0.05, query coverage >= 80%). The network was generated from Cytoscape with Organic layout. Most sequences form two groups: one contains PYP from cluster 7 (shown in yellow), and the other contains PAS domains with diverse functions (shown in red, blue, and green). Yellow, PYP homologs; red, heme-binding PAS domains; blue, FAD-binding PAS domains; green, FMN-binding PAS domains; grey, other ligand-binding PAS domains. **B.** Sequence logos for the two groups from panel A: PYP (top) and other PAS domains (bottom). Asterisks show conserved residues that distinguish PYP from other PAS domains. **C.** Conserved aromatic amino acids in PYP pocket. Yellow, pCA; blue, phenylalanines; red, tyrosines.

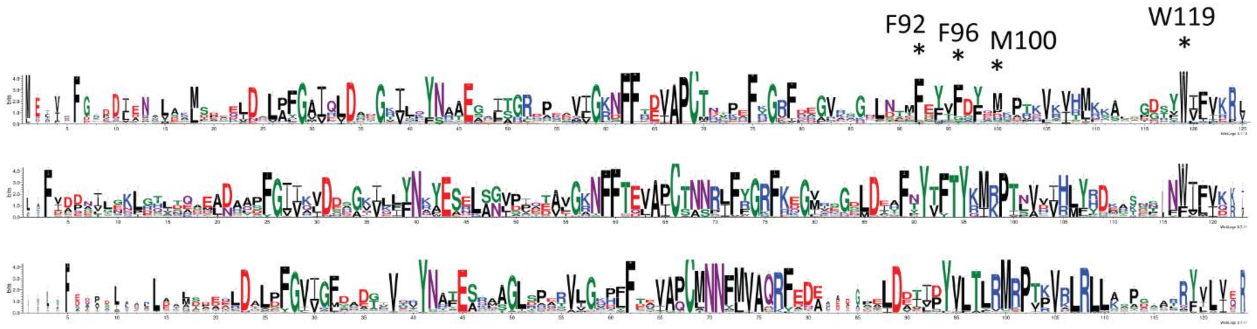

Fig. S2. Sequence logos of Clades A, B and C (from top to bottom). PYP homologs with C69 substitutions were not included for alignments since they are distantly related to PYP and may have different functions. Gaps were trimmed at the 90% level. Asterisks show different conserved residues between Clades A, B, and C.

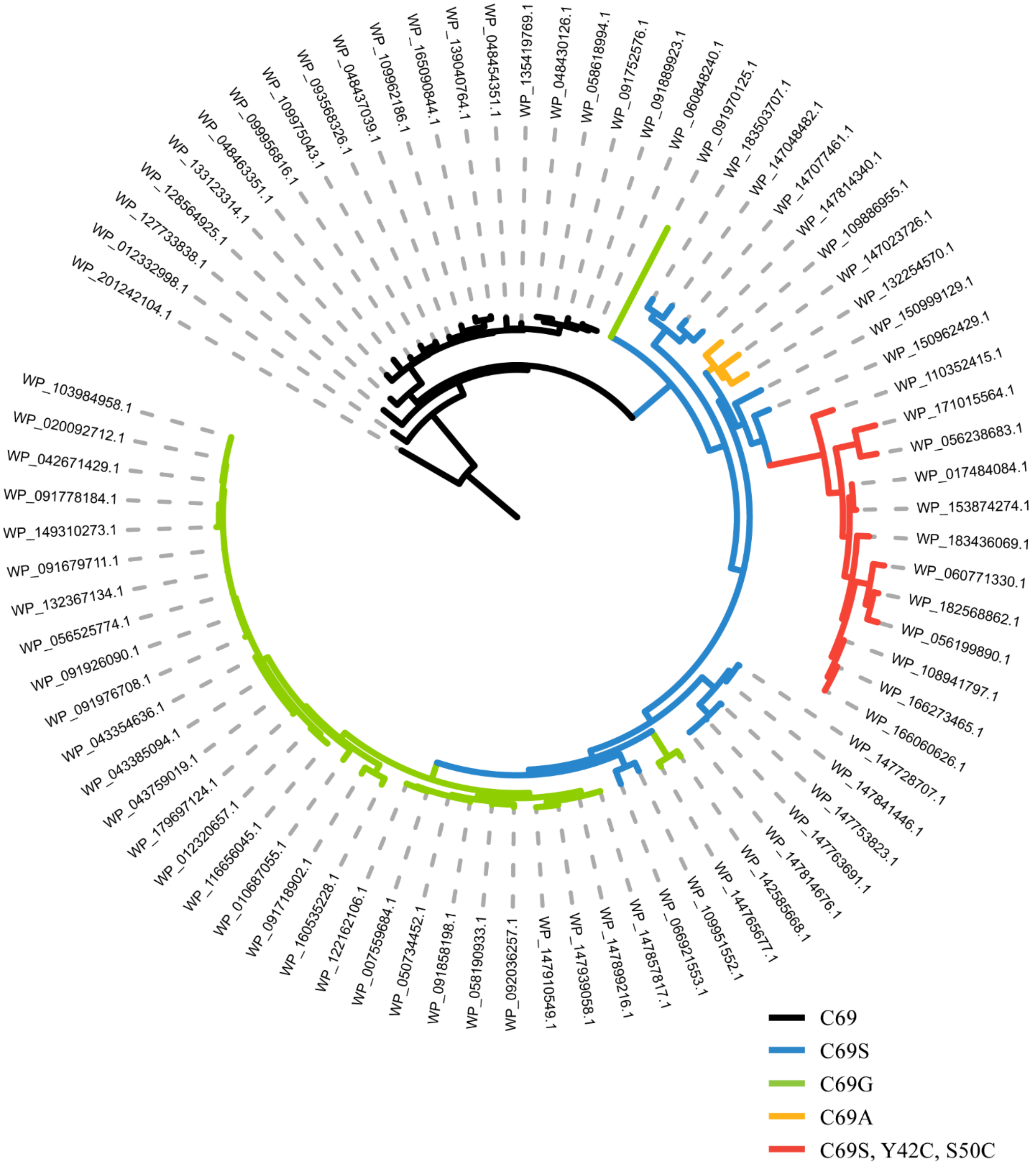

Fig. S3. Maximum likelihood tree of methylobacterial Ppr-PYP. Sequences were obtained by a BLAST search within the Methylobacterium genus using *H. halophila* PYP (NCBI accession: WP_201242104.1) as the query. *H. halophila* PYP (NCBI accession: WP_201242104.1) was used as the outgroup to root the tree. Black, PAS with conserved C69; blue, PAS with C69S mutation; green, PAS with C69G mutation; orange, PAS with C69A mutation; red, PAS with C69S, Y42C, and S50C mutations.

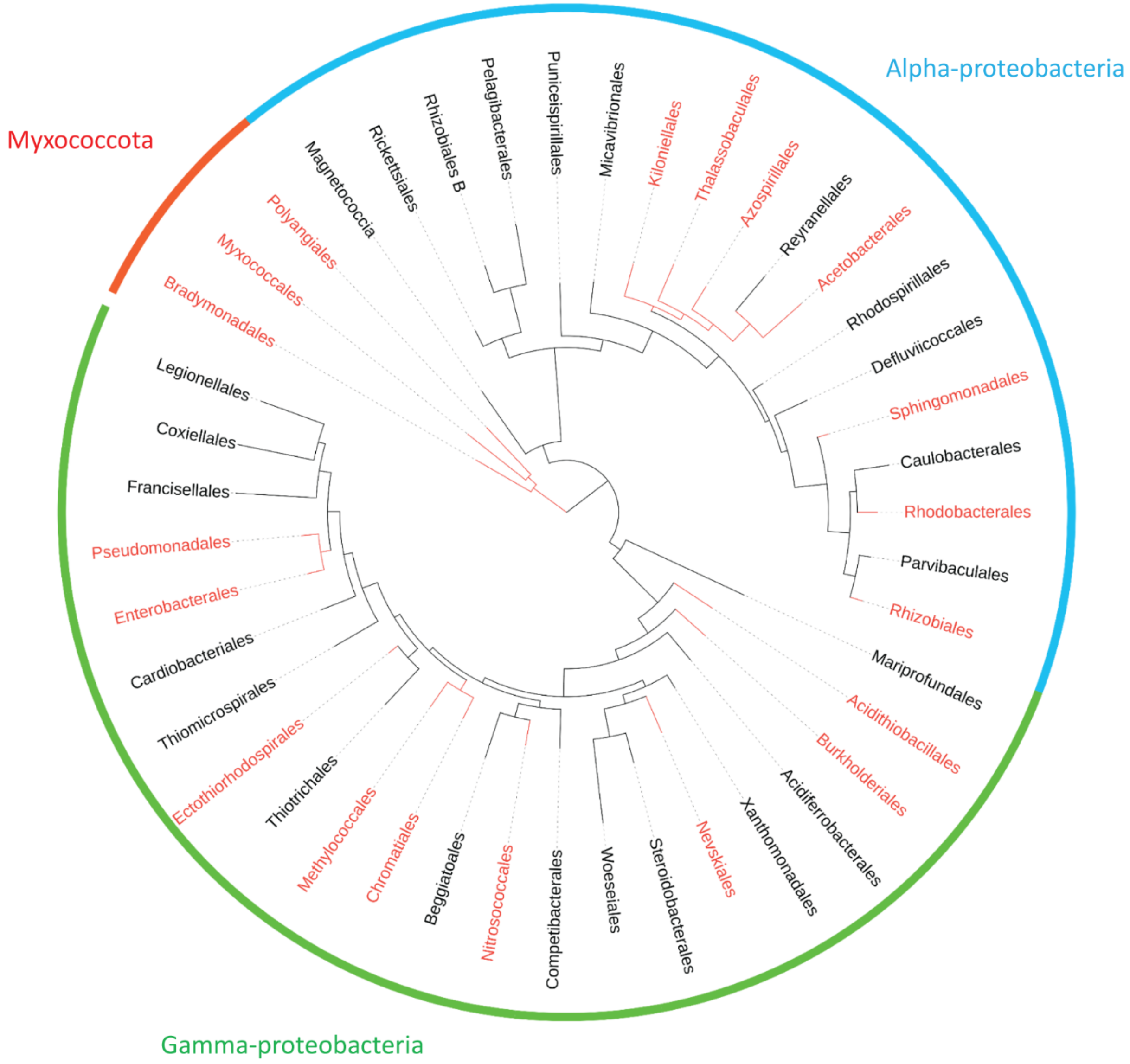

Fig. S4. Phylogenetic tree of Myxococcota and Proteobacteria orders based on AnnoTree. Bacterial orders with at least 10 genomes were shown. Red, bacterial orders with at least one genome containing functional PYP.

Table S1 Members of the PAS superfamily in Pfam

Pfam Family	Pfam Entry	InterPro Entry	Number of Domains
PAS**	PF00989	IPR013767	58,225
PAS_4	PF08448	IPR013656	58,162
PAS_3	PF08447	IPR013655	57,710
PAS_9	PF13426	IPR000014	38,837
PAS_8	PF13188	IPR000014	16,764
MLTR_LBD	PF17765	IPR041413*	10,563
PAS_7	PF12860	-	8,171
PAS_11	PF14598	-	7,663
PAS_2	PF08446	IPR013654	3,281
PAS_10	PF13596	-	2,777
MEKHLA	PF08670	IPR013978*	1,797
PAS_6	PF08348	IPR013559*	1,657
PAS_5	PF07310	IPR009922*	1,337
CpxA_peri	PF16527	IPR032404*	194
DUF5593	PF18007	IPR041458*	157
AbfS_sensor	PF18225	IPR041124*	23
PAS_12	PF18095	IPR040578*	15
**Total (PAS superfamily)**	**CL0183**	**IPR035965**	**267,333**

* Families removed from the PAS superfamily in InterPro.

** Family used for clustering.

Table S2 Markov Clustering with different inflation parameter values

Inflation	Number of sequences in each cluster											
1.0	Failed											
1.2	32,034	1,522	81									
1.3	26,067	4,412	1,512	918	355	94	88	84	62	36			
1.4	25,890	4,380	1,519	930	355	180	95	87	86	63	36	16	
2.0	23,774	4,682	1,517	975	477	451	383	344	252	183	95	87	81
	65	56	39	37	27	20							
6.0	1,952	1,875	1,611	1,434	1,381	975	566	426	317	313	279	247	238
	226	198	175	156	141	123	116	112	98	94	90	88	79
	77	76	75	72	71	71	69	69	68	67	59	58	58
	55	55	54	53	49	46	43	42	42	38	36	35	33
	32	32	31	31	31	31	30	29	28	27	26	25	25
	23	22	22	21	20	17	17	17	17	17	16	16	16

Clusters containing PYP under each inflation are highlighted in red. Clusters with 15 or fewer sequences are not shown for simplicity.

Table S3 Ligand/cofactor-binding PAS domains used for all-vs-all BLAST

Organism	Protein	UniProt Accession	PAS Location	Pfam Family	Ligand/ Cofactor	PDB Accession*
Bradyrhizobium diazoefficiens	FixL	FIXL_BRADU	143-256	PAS	Heme	1DP6
Rhizobium meliloti	FixL	FIXL_RHIME	137-250	PAS	Heme	1D06
Escherichia coli	DosP	DOSP_ECOLI	22-126	PAS_9	Heme	1V9Z
Pseudomonas aeruginosa	Aer2	DOSP_ECOLI	22-126	PAS_8	Heme	4HI4
Vibrio cholerae	Aer2	Q9KKL2_VIBCH	175-281	PAS_9	Heme	6CEQ
Pseudomonas aeruginosa	BdlA	BDLA_PSEAE	8-111	PAS_9	Heme	-
Homo sapiens	CLOCK	CLOCK_HUMAN	120-260	PAS	Heme	-
Homo sapiens	NPAS2	NPAS2_HUMAN	95-235	PAS	Heme	-
Azospirillum brasilense	AerC	WP_145689077.1 (not found in UniProt)	25-131, 146-252	PAS_9, PAS_3	FAD, FAD	-
Escherichia coli	Aer	AER_ECOLI	10-115	PAS_3	FAD	-
Methylococcus capsulatus	MmoS	Q609M8_METCA	97-200	PAS_9	FAD	3EWK
Azotobacter vinelandii	NifL	NIFL_AZOVI	25-136	PAS	FAD	2GJ3
Neurospora crassa	Vivid	Q1K5Y8_NEUCR	70-182	PAS_9	FAD	3D72
Neurospora crassa	Vivid	Q9C3Y6_NEUCS	70-182	PAS_9	FAD	2PD7
Phaeodactylum tricornutum	LOV	A0A140UHJ0_PHATR	14-118	PAS_9	FMN	5A8B
Dinoroseobacter shibae	LOV	A8LP63_DINSH	35-137	PAS_9	FMN	4KUK
Vaucheria frigida	LOV	A8QW55_VAUFR	217-322	PAS_9	FMN	3UE6
Oryza sativa	LOV	ADO1_ORYSJ	57-169	PAS_9	FMN	-
Phaeodactylum tricornutum	LOV	B7G9J2_PHATC	1-100	PAS_9	FMN	5DKK
Chloroflexus aggregans	LOV	B8GAY9_CHLAD	48-152	PAS_9	FMN	6RHF
Ochromonas danica	LOV	C5NSW6_OCHDN	194-298	PAS_9	FMN	6I20
Nakamurella multipartita	LOV	C8XJT7_NAKMY	246-352	PAS_9	FMN	6HMJ
Hypocrea jecorina	LOV	G0RUC2_HYPJQ	98-204	PAS_9	FMN	4WUJ
Avena sativa	LOV	O49003_AVESA	413-518	PAS_9	FMN	2V0U
Avena sativa	LOV	O49004_AVESA	416-521	PAS_9	FMN	5DJT
Pseudomonas putida	LOV	Q88E39_PSEPK	15-121	PAS_9	FMN	2YOM
Adiantum capillus-veneris	LOV	Q9ZWQ6_ADICA	929-1032	PAS_9	FMN	1G28
Brucella abortus	LOV	LOVHK_BRUA2	31-137	PAS_9	FMN	5EPV
Brucella melitensis	LOV	LOVHK_BRUME	31-137	PAS_9	FMN	3T50
Arabidopsis thaliana	LOV	PHOT1_ARATH	198-302, 475-580	PAS_9	FMN	2Z6C

Arabidopsis thaliana	LOV	PHOT2_ARATH	134-238, 388-494	PAS_9	FMN	2Z6D
Bacillus subtilis	LOV	PHOT_BACSU	24-128	PAS_9	FMN	2MWG
Chlamydomonas reinhardtii	LOV	PHOT_CHLRE	21-125	PAS_9	FMN	1N9L
Staphylococcus carnosus	NreB	NREB_STACT	3-137	-	Fe-S cluster	-
Haloarcula marismortui	Q5V5P7	Q5V5P7_HALMA	406-513	PAS_4	indole-3-carbaldehyde	3BWL
Halorhodospira halophila	PYP	PYP_RHOCB	19-121	PAS	*p*-coumaric acid	1NWZ
Vibrio cholerae	VqmA	Q9KKM5_VIBCH	86-198	PAS_4	DPO	6KJU
Vibrio cholerae	VqmA	A0A0F0AZW0_VIBCL	13-126	PAS_4	DPO	6UGL
Pseudomonas putida	TodS	TODS_PSEP1	43-154	PAS_4	Monoaromatic ligands	5HWT
Staphylococcus aureus	WalK	WALK_STAAU	80-175	PAS_9	Zn	4MN5
Cronobacter turicensis	RpfR	C9XTL5_CROTZ	141-244	PAS_9	Lauric acid	6DGA
Mycobacterium tuberculosis	Rv1364c	MTDRP_MYCTU	21-135	PAS_4	Palmitic acid	3K3C
Rhodococcus jostii	RHA05790	Q0S7I6_RHOJR	124-226	PAS_4	Butanoic acid	3FG8
Homo sapiens	AHR	AHR_HUMAN	277-383	PAS_3	Aromatic ligands	-

* For each protein, a PDB structure with ligand/cofactor-binding is listed (if available).
